# Radioprotective Effect of Hesperidin: A Systematic Review

**DOI:** 10.3390/medicina55070370

**Published:** 2019-07-12

**Authors:** Ahmed Eleojo Musa, Gilnaz Omyan, Farid Esmaely, Dheyauldeen Shabeeb

**Affiliations:** 1 Department of Medical Physics, Tehran University of Medical Sciences (TUMS), Tehran 1416753955, Iran; 2 Department of Physics, University of Guilan, Guilan 43714, Iran; 3 Radiotherapy and Oncology Research Center, Cancer Institute, TUMS, Tehran 1416753955, Iran; 4 Misan Radiotherapy Center, Misan Health Directorate, Ministry of Health/Environment, Misan 62010, Iraq; 5 Department of Physiology, College of Medicine, University of Misan, Misan 62010, Iraq

**Keywords:** Hesperidin, radiation protection, radiotherapy, DNA damage, ionizing radiation

## Abstract

*Background and objectives*: Ionizing radiation (IR) has been of immense benefit to man, especially for medical purposes (diagnostic imaging and radiotherapy). However, the risks of toxicity in healthy normal cells, leading to cellular damage as well as early and late side effects, have been major drawbacks. The aim of this study was to evaluate the radioprotective effect of hesperidin against IR-induced damage. *Materials and Methods:* The preferred reporting items for systematic reviews and meta-analyses (PRISMA) were applied in reporting this study. A search was conducted using the electronic databases PubMed, Scopus, Embase, Google Scholar, and www.ClinicalTrials.gov for information about completed or ongoing clinical trials. *Results:* From our search results, 24 studies involving rats, mice, and cultured human and animal cells were included. An experimental case—control design was used in all studies. The studies showed that the administration of hesperidin reduced oxidative stress and inflammation in all investigated tissues. Furthermore, it increased 30-day and 60-day survival rates and protected against DNA damage. The best radioprotection was obtained when hesperidin was administered before irradiation. *Conclusions:* The results of the included studies support the antioxidant, anti-inflammatory, and antiapoptotic abilities of hesperidin as a potential radioprotective agent against IR-induced damage. We recommend future clinical trials for more insights.

## 1. Introduction

Since the discovery of ionizing radiation (IR) in the early 1900s, its use has been on the increase. Nowadays, the use of IR can be found in industrial and agricultural sectors. However, it is mostly utilized in medicine for diagnostic as well as treatment aims. The use of IR for diagnostic purposes is responsible for the majority of radiation doses received by man [1,2]. Moreover, radiotherapy (cancer treatment using IR) is more utilized compared to other cancer therapeutic modalities [3].

Despite the numerous benefits of IR to man, the risks of toxicity to healthy normal cells leading to cellular damage as well as early and late side effects have been major drawbacks. IR causes damage to cells via direct and indirect effects. Direct effects occur when IR interacts directly with DNA. Indirect effects take place as a result of the interaction between free radicals, including reactive oxygen species (ROS) and reactive nitrogen species (RNS), and neighboring DNA molecules [4]. The most important biological molecule in the cell for sustaining life, DNA, is the primary target for radiation-induced cell death.

In addition to radiation exposure for medical purposes, several side effects due to radiation exposure from nuclear accidents as well as radiation disasters have been reported. The Hiroshima and Nagasaki events during World War II led to the deaths of over 150,000 people who were exposed to sublethal radiation doses [5]. The Chernobyl nuclear disaster was also another event that led to chronic biological changes to the immune system as well as subsequent cancer development in exposed persons [6].

Most radiation-induced side effects are due to the free radicals produced by IR in cells [7]. Radioprotectors (or radioprotective agents) have been proposed for preventing or reducing these side effects. A radioprotector is most suitable for use if it has minimal toxicity while protecting healthy cells and not cancer cells [8]. It is also important that they are easily accessible and not expensive [9]. The use of amifostine, the first Food and Drug Administration (FDA)-approved radioprotector, has been mostly restricted as a result of possible toxicity [10]. Therefore, recent studies on radioprotectors have been majorly centered on natural substances such as flavonoids, due to their minimal side effects.

Flavonoids of varying phenolic structures are present in natural substances with varying phenolic structures such as fruits, vegetables, grains, bark, roots, stems, flowers, tea, and wine [11]. It has been shown that the potent antioxidant effects of flavonoids are a result of their high redox abilities, making them efficient hydrogen donors and reducing agents, in addition to their metal-chelating capabilities and singlet oxygen quenchers [12]. Some flavonoids that have been explored for radioprotection include curcumin, sesamol, hesperidin, rutin, ocimum sanctum, quercetin, and resveratrol [13].

**Figure 1:**
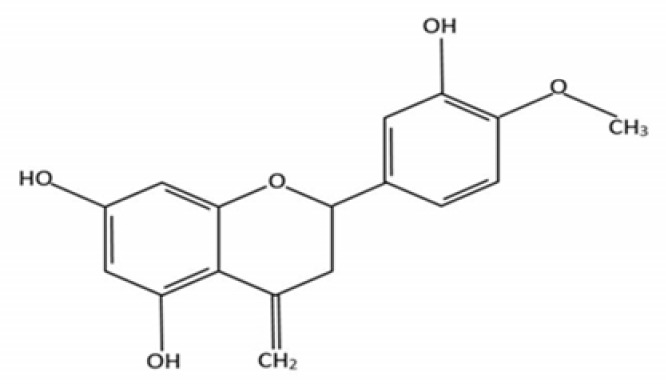
Chemical structure of hesperidin.

Hesperidin (hesperetin-7-rhamnoglucoside, Figure 1) is a bioflavonoid found in citrus fruits such as tangerine, orange, and lemon as well as in plant extracts such as tea and olive oil. Citrus fruit peels have the highest concentrations of hesperidin. It has shown promising results in the treatment of inflammatory as well as allergy diseases [14]. Its potential in the treatment of cardiovascular and neurological disorders has also been investigated [15,16]. Studies have shown that hesperidin possesses antimicrobial, anticarcinogenic, antioxidant, and anti-inflammatory effects and decreased capillary fragility [17]. The purpose of this systematic review was to evaluate the radioprotective effect of hesperidin against radiation-induced damage to cells and organs.

## 2. Materials and Methods

### 2.1. Search Strategy

The reporting of this systematic review was done according to the statement of preferred reporting items for systematic reviews and meta-analyses (PRISMA) [18]. A computer-based literature search was conducted in January 2019 using PubMed, Scopus, Embase, and Google Scholar for articles published in English. No limit in publication year was applied. The following keywords were used for our literature search: “hesperidin”, “radiation”, “radiation protection”, and “radioprotector”. We also searched www.ClinicalTrials.gov for completed or ongoing clinical trials. In addition, references of retrieved studies were manually screened to obtain relevant studies.

### 2.2. Inclusion Criteria

The articles retrieved were based on the following inclusion criteria:

- Studies that were conducted to determine the radioprotective effect of hesperidin and were published in the English language;
- Studies in which ionizing radiation was used; and
- Experimental and clinical studies with full texts.

### 2.3. Exclusion Criteria

We excluded studies based on the following criteria:

- Studies in which hesperidin was not used;
- Studies in which hesperidin was used in combination with other agents;
- Studies that made use of other forms of radiation such as ultraviolet (UV), fluorescence, cosmic, etc.;
- Studies that evaluated the effect of hesperidin with chemotherapy instead of radiation therapy; and
- Conference abstracts, simulation studies, review articles, case reports, letters, editorials, unpublished data, articles without full texts, and non-English articles.

### 2.4. Study Selection

All retrieved articles from electronic as well as manual searches were entered into endnote software (EndNote version X6, Thomson Reuters, New York, NY, USA). Thereafter, duplicates were removed. Afterwards, two authors (A.E.M. and G.O.) independently reviewed the titles and abstracts of the retrieved studies for eligibility. Studies were then selected based on the predetermined inclusion and exclusion criteria. For any disagreements concerning the inclusion of studies, all authors agreed on a consensus based on factual evidence.

### 2.5. Data Extraction

Data from each eligible study were extracted by A.E.M. and G.O. and checked by F.E. and D.S. The following information was obtained: Author name, year of publication, subject, organ (or tissue) of interest, radiation type and dose, hesperidin dose, as well as time for outcome assessment. Furthermore, the main outcomes were summarized and included.

## 3. Results

### 3.1. Literature Search

**Figure 2:**
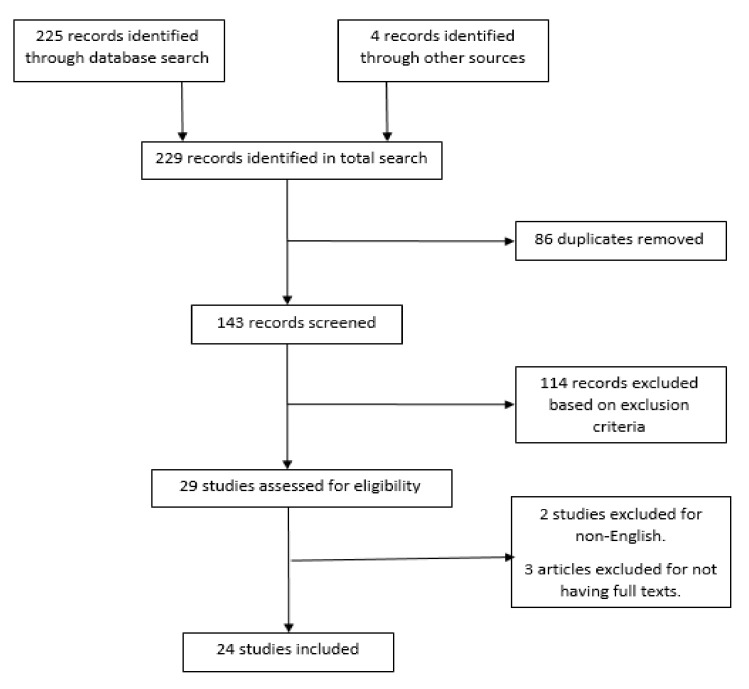
Preferred reporting items for systematic reviews and meta-analyses (PRISMA) flow diagram for the selection of included articles.

The PRISMA flow diagram showing our search results is presented in Figure 2. Our initial search gave a total of 229 records, with the breakdown as follows: 225 records from electronic databases and 4 records obtained through a manual search. Our search of the online database of www.ClinicalTrials.gov showed that there were no completed or ongoing clinical trials evaluating the radioprotective effect of hesperidin. From these figures, 143 records were retained after removing duplicates. Following careful examination and screening of their titles and abstracts as well as the application of the inclusion and exclusion criteria, a further 114 records were excluded. The full texts of the remaining 29 records were assessed. We excluded two articles for non-English language publication, while three more records were removed for not having full texts. Finally, a total of 24 studies were included in this systematic review.

### 3.2. Study Characteristics

The summary of data showing the characteristics of included studies is presented in Table 1. These articles, published between 2006 and 2018, employed an experimental case–control design. Furthermore, they include 4 in vitro, 17 in vivo, 1 in vitro/in vivo, and 2 in vivo/in vitro studies using rats, mice, and cultured human and animal cells. Gamma (γ)-radiation was utilized in 21 studies (with 1 study making use of a γ-ray from Technetium sestamibi (^99m^Tc-MIBI) radiopharmaceuticals), and X-ray radiation was used in 3 studies. The doses of the radiation were between 1 and 18 Gy. Hesperidin was administered orally in 18 studies and intraperitoneally in 2 studies.

### 3.3. Hesperidin Dosage

A hesperidin dose of 100 mg/kg body weight was mostly used in the included studies to assess its radioprotective effect. In addition, this dose has been shown to be the most effective in reducing the healing time of radiation-induced wounds by two days [24]. Results from another study by Haddadi et al. also showed that this same oral dose of hesperidin was effective in accelerating wound healing from radiation-induced skin damage [31].

Different effective hesperidin doses have also been reported in several studies. In a study by Hosseinimehr and Nemati, a hesperidin dose of 80 mg/kg showed a maximum reduction in the frequencies of micronucleated polychromatic erythrocytes (MnPCEs) [30]. In a later study by Hosseinimehr et al., they observed that maximum radioprotection was obtained 1 h after oral ingestion of 250 mg/kg hesperidin [20].

Kalpana et al. detected maximum protection against radiation-induced reproductive death for a hesperidin concentration in a cell medium of 16.38 μM (9.99 mg/L) [21]. In another study, 25 mg/kg hesperidin was the most effective dose, restoring antioxidant status to normal levels as well as decreasing lipid peroxidation and preventing DNA damage [28]. Furthermore, Shaban et al. showed that the best radioprotection by hesperidin was obtained when administered before irradiation [33].

### 3.4. Toxicity and Survival Analysis

In all included studies, there was no reported case of toxicity or side effects following the administration of hesperidin. In an in vivo/in vitro study by Hosseinimehr et al., five human subjects each received a hesperidin dose of 250 mg/kg orally before collection of their blood samples. They showed no adverse signs 0 (before), 1, 2, and 3 h post-hesperidin ingestion [20].

In a study by Rezaeyan et al., rats treated with 100 mg/kg hesperidin before exposure to an 18-Gy single-dose X-ray showed significant improvement in survival rates compared to those in the radiation group (with a median survival period of 55 days). A 60-day follow-up indicated that five rats survived in the radiation group, while eight survived in the hesperidin-pretreated group [38].

Hesperidin also improved survival in a study investigating its protective effect against radiation-induced lung damage. Rats were administered 100 mg/kg hesperidin orally for seven consecutive days before exposure to 18-Gy γ-rays. After a 60-day period of observation, 4 out of 10 rats in the radiation group survived, while 7 out of 10 rats survived in the hesperidin + radiation group [32].

In evaluating the radioprotective effect of hesperidin against radiation-induced hepatic damage, Kalpana et al. showed that mice pretreatment with 25 mg/kg hesperidin before exposure to 10-Gy γ-rays increased the median survival period to 15 days compared to the nontreated groups (6 days) exposed to the same radiation dose. Daily monitoring of these rats was done for 30 days [28].

## 4. Discussion

Exposure to IR can affect cellular components of living tissues, leading to early and late side effects. The severity of IR-induced complications on normal tissues varies with radiation dose as well as cell or organ type [43]. Early effects such as apoptosis, lymphocyte adhesion and infiltration, vascular permeability, increased endothelial cell swelling, and edema occur within hours after radiation exposure [44]. Late effects including necrosis, organ dysfunction, death, cancer, etc., occur months to years following exposure [45]. These radiation effects pose serious concerns to humans, especially to children, who are more radiosensitive [46]. If adequate protective measures are not put in place, they may also negatively impact the quality of life of patients exposed to either diagnostic or therapeutic doses of IR.

One of the strategies for countering radiation-induced damage is the use of radioprotectors. The efficacy of natural radioprotective agents such as hesperidin has been explored in recent times. Thus, in the present study, using a systematic review design, we searched for studies that made use of hesperidin as a radioprotective agent against IR-induced damage.

Results from the included studies showed that hesperidin demonstrated a protective effect against both gamma and X-rays in the healthy tissue of mice, rats, and cultured human and animal cells (Table 1). These effects were observed in hesperidin doses as low as 10 mg/kg administered orally. Furthermore, hesperidin treatment reduced biochemical markers of oxidative stress and improved histopathological outcomes of exposed tissues [27]. Moreover, its antiapoptotic activities were demonstrated in mouse testes exposed to IR [33].

Skin, the body’s largest organ, is inevitably exposed to IR during radiotherapy. It has been observed that bone marrow and skin epithelia have high susceptibility to IR-induced side effects [47]. It has been estimated that 90–95% of patients who receive radiotherapy show varying grades of radiation-induced skin reactions [48,49]. Skin complications arising after radiotherapy are referred to as radiodermatitis. Radiodermatitis is a common side effect after radiotherapy for breast cancer. Furthermore, acute radiodermatitis, with indications including scaling, edema erythema, erosion, and ulcers, could arise 90 days after radiotherapy. Chronic radiodermatitis can be observed a few months after radiotherapy, with symptoms such as changes in skin texture, hypopigmentation or hyperpigmentation, teleangiectasis, and poikiloderma. Hesperidin has been shown to prevent radiation-induced skin burns via initiating the formation of new vessels and a microvascular network through vascular endothelial growth factor (*VEGF*) gene induction [31].

Cardiovascular diseases are the leading causes of mortality worldwide [50]. It has been projected that by the year 2030, cardiovascular diseases will account for an annual mortality of more than 23.3 million people [51]. The risk of radiation-induced heart diseases increases with IR doses to heart tissues after radiotherapy for lung, breast, or esophageal cancers (due to the close proximities of these organs to the heart), as well as in nuclear disasters. The functionalities of coronary vessels, valves, the pericardium, and the myocardium are adversely affected after irradiation [52]. Our investigation of the included studies showed potent radioprotective and anti-inflammatory effects of hesperidin on heart tissues. The administration of hesperidin before irradiating the thorax with a high dose of gamma radiation showed an improvement in survival as well as a reduction in oxidative damage, vascular leakage, inflammation, the fibrosis and infiltration of macrophages, lymphocytes, and mast cells [38]. It was also effective in ameliorating serum heart disease markers [42] and preventing cellular damage to the heart [40]. Similar outcomes were obtained for lung tissues [32,35,36].

Promising radioprotective effects have been observed for some natural antioxidants, such as melatonin, selenium, Coenzyme Q10, α-tocopherol, caffeic acid, and ascorbic acid. [13]. Various clinical trials have also confirmed the efficacy of some of these natural products [53,54]. Since experimental findings have shown encouraging radioprotective results for hesperidin, it would be interesting to see how it competes with other radioprotectors. 

The present review has some limitations. First, we reviewed studies that used animals as well as cultured human and animal cells due to the nonavailability of clinical trials. Furthermore, in the studies included, the radioprotective effect of hesperidin was only investigated in healthy tissues. It would be desirable to observe these effects in tumor cells.

## 5. Conclusions

In the results of the included studies in this review, it was shown that hesperidin has the potential to be an effective radioprotector against IR-induced damage to cellular components of healthy tissues. It showed promising antioxidant, anti-inflammatory, and as antiapoptotic abilities. There were no reported side effects due to its administration. We suggest future clinical trials to further assess its efficacy as well as its optimal dose. This is necessary in order to assess the clinical effects of experimental melatonin doses as well as for more insight into possible variations between experimental outcomes using cells or animals and those in humans. Comparative studies with other radioprotectors will be required in order to investigate its effectiveness. The effects of hesperidin treatment on cancer cells exposed to ionizing radiation should also be investigated. Lastly, more studies of the molecular mechanisms of radioprotection by hesperidin would be desirable.

## Figures and Tables

**Table 1:** Summary of articles included in the systematic review.

First Author	Subject	Organ (or Tissue) of Interest	Radiation Type and Dose (Gy)	Hesperidin Dose/Concentration	Time for Outcome Assessment	Main Outcomes
Katoch et al. [19]	Cultured human fibroblast cells	Fibroblast cells	γ-ray, 5	6.18 ± 0.26 mg/g extract	4 h	Countered radiation-induced free radicals post-irradiation, decreased prolonged oxidative stress, and protected against radiation-induced DNA damage.
Hosseinimehr et al. [20]	Cultured human blood lymphocytes	Lymphocytes	γ-ray, 1.5	250 mg/kg body weight	0–3 h	Significant decrease in the incidence of micronuclei of blood lymphocytes collected 1 h after oral administration of hesperidin compared to those collected at 0 h. Maximum protection and decrease in frequency of micronuclei (33%) was observed at 1 h after ingestion of hesperidin.
Kalpana et al. [21]	Cultured human lymphocytes	Lymphocytes	γ-ray, 1–4	3.27–19.65 µM	30 min	Here, 16.38 µM hesperidin pretreatment prior to irradiation had the maximum radioprotective effect, which included a significant decrease in the levels of MN and DC counts, as well as TBARS. Reduction in tail length, tail moment, olive tail moment, and % DNA in the tail. Increased levels of enzymatic (SOD, CAT, and GPx) and non-enzymatic (glutathione (GSH)) antioxidants and restored DNA damage to near-normal levels.
Hosseinimehr et al. [22]	Cultured human lymphocyte cells	Lymphocytes	γ-ray from ^99m^Tc-MIBI radiopharmaceuticals, 200 μCi	10–100 μM	3 h	Significant reduction in micronuclei frequency in cultured lymphocytes, thereby leading to protection against genetic damage. Optimal effect of hesperidin was obtained at 100 μM concentration.
Kang et al. [23]	Cultured BALB/c mice splenocytes	Splenocytes	γ-ray, 2 and 4	20–500 µM	24 h	Improved cell viability, prevented damage to DNA, and hindered proinflammatory cytokines, intracellular ROS, and NO.
Jagetia et al. [24]	Swiss albino mice	Skin wound	γ-ray, 6	0–500 mg/kg body weight	3–15 days	Treatment with 100 mg/kg hesperidin before irradiation had the maximum radioprotective effect, leading to a steady increase in wound contraction and reduction in mean wound healing time by 2 days.
Jagetia et al. [25]	Swiss albino mice	Skin wound	γ-ray, 2–8	100 mg/kg body weight	1–15 days	Enhancement of collagen, hexosamine, DNA, and nitric oxide synthesis in the granulation tissue, thereby improving wound healing compared to the irradiated group.
Jagetia et al. [26]	Swiss albino mice	Skin wound	γ-ray, 2–8	100 mg/kg body weight	1–15 days	Significantly reduced both radiation-induced delay in wound contraction and mean wound healing time.
Jagetia et al. [27]	Swiss albino mice	Skin wound	γ-ray, 6	50 and 100 mg/kg body weight	0–48 h	Reduced radiation-induced oxidative stress in the irradiated wounds of mice.
Kalpana et al. [28]	Swiss albino mice	Liver	X-ray, 4	12.5–100 mg/kg body weight	30 days	Here, 25 mg/kg hesperidin pretreatment prior to irradiation had the maximum radioprotective effect, including restoring antioxidant status to near-normal as well as decreasing the levels of the lipid peroxidation index, DNA damage, and comet parameters.
Lee et al. [29]	ICR mice	Liver, intestine, splenocytes, and lymphocytes	X-ray, 15	50 and 200 mg/kg body weight	10 and 30 days	Reduction of radiation-induced inflammation and partial restoration of immune and nutritional status.
Hosseinimehr et al. [30]	NMRI mice	Bone marrow cells	γ-ray, 2	10–160 mg/kg body weight	24 h	Hesperidin dose of 80 mg/kg had the maximum reduction in the frequencies of MnPCEs. Significant increase in PCE/PCE + NCE ratio in mice bone marrow compared to nondrug-treated irradiated control.
Haddadi et al. [31]	Sprague-Dawley rats	Skin	γ-ray, 22	100 mg/kg body weight	24 h	Initiated angiogenesis by inducing *VEGF* gene. Stimulated epithelialization and collagen deposition and enhanced cellular proliferation, thereby aiding wound healing and protecting skin from radiation damage.
Haddadi et al. [32]	Sprague-Dawley rats	Lung	γ-ray, 18	100 mg/kg body weight	24 h and 8 weeks for acute and chronic histopathological evaluations, respectively.	Hesperidin administration led to significant decrease in radiation-induced inflammation and inflammatory cells at 24 h post-irradiation. Furthermore, there was a reduction in radiation pneumonitis and radiation fibrosis in the lung tissue at 8 weeks post-irradiation.
Shaban et al. [33]	Sprague-Dawley rats	Testes	γ-ray, 8	200 mg/kg body weight	8 and 14 days	Reduction in OS, LPO, and apoptosis. Improvement in structure of testes and better protection of testes was observed when hesperidin was administered before irradiation compared to after irradiation.
Karimi et al. [34]	Rats	Lens	γ-ray, 15	100 mg/kg body weight	2 days	Significant increase in the GSH level and decrease in MDA level, and hence, a reduction in oxidative stress.
Abd El-Rahman et al. [35]	Albino rats	Blood, lung, and dorsal aorta	γ-ray, 6	40 mg/kg body weight	21 days	Significantly reduced lipid variation, decreased oxidative stress, improved blood cell counts, and attenuated lung and dorsal aorta tissue injury.
Rezaeyan et al. [36]	Rats	Lung	γ-ray, 18	100 mg/kg body weight	24 h	Significant reduction in macrophages and neutrophils, as well as mild reduction in inflammation and lymphocytes.
Fardid et al. [37]	Rats	Peripheral blood lymphocytes	γ-ray, 2 and 8	50 and 100 mg/kg body weight	24 h	Pretreatment with hesperidin significantly reduced apoptosis in irradiated rats.
Rezaeyan et al. [38]	Rats	Heart	X-ray, 18	100 mg/kg body weight	24 h (for biochemical assay and acute histopathological evaluation) and 8 weeks (for chronic histopathological evaluation)	Decreased inflammation, fibrosis, mast cell, and macrophage numbers and myocyte necrosis.
Ahmed et al. [39]	Albino rats	Bone	γ-ray, 2	160 mg/kg body weight	24 h	Improvement in antioxidant activities as well as biomechanical properties of bone and prevention of endothelial dysfunction.
Pradeep et al. [40]	Sprague-Dawley rats	Liver, heart, and kidney	γ-ray, 5	50 and 100 mg/kg body weight	7 days	Reduction in necrotic and cellular damage, as well as oxidative stress.
Said et al. [41]	Albino rats	Brain	γ-ray, 5	50 mg/kg body weight	14 days	Significant reduction in oxidative stress, monoamine alterations, and mitochondrial damage, and hence a reduction in the severity of radiation-induced biochemical brain disorders.
Park et al. [42]	Sprague-Dawley rats	Heart and kidney	γ-ray, 5	50 and 100 mg/kg body weight	7 days	Treatment with hesperidin post-irradiation led to significant reduction in levels of lipid peroxidation, improvements in activities of endogenous antioxidants (SOD, CAT, GPx, and GSH), and minimal damage to the heart and kidney tissues.

OS: Oxidative stress, LPO: Lipid peroxidation, GSH: Glutathione, MDA: Malondialdehyde, ROS: Reactive oxygen species, NO: Nitric oxide, VEFG: *Vascular endothelial growth factor*, MN: Micronuclei, DC: Dicentric aberration, ^99m^Tc-MIBI: Technetium sestamibi, ICR: Institute of Cancer Research, NMRI: Naval Medical Research Institute, BALB: Bagg Albino, TBARS: Thiobarbituric acid reactive substances, SOD: Superoxide dismutase, CAT: Catalase, GPx: Glutathione peroxidase, MnPCEs: Micronucleated polychromatic erythrocytes, and NCE: Normochromatic erythrocyte.

## References

[1] De Gonzalez A.B., Darby S. (2004). Risk of cancer from diagnostic X-rays: Estimates for the UK and 14 other countries. Lancet.

[2] De González A.B., Mahesh M., Kim K.-P., Bhargavan M., Lewis R., Mettler F., Land C. (2009). Projected cancer risks from computed tomographic scans performed in the United States in 2007. Arch. Intern. Med..

[3] Ringborg U., Bergqvist D., Brorsson B., Cavallin-Ståhl E., Ceberg J., Einhorn N., Frödin J.-E., Järhult J., Lamnevik G., Lindholm C. (2003). The Swedish Council on Technology Assessment in Health Care (SBU) systematic overview of radiotherapy for cancer including a prospective survey of radiotherapy practice in Sweden 2001—Summary and conclusions. Acta Oncol..

[4] Wang H., Mu X., He H., Zhang X.-D. (2018). Cancer radiosensitizers. Trends Pharmacol. Sci..

[5] Shimizu Y., Kodama K., Nishi N., Kasagi F., Suyama A., Soda M., Grant E.J., Sugiyama H., Sakata R., Moriwaki H. (2010). Radiation exposure and circulatory disease risk: Hiroshima and Nagasaki atomic bomb survivor data, 1950–2003. BMJ.

[6] Berger E.M. (2010). The Chernobyl disaster, concern about the environment, and life satisfaction. Kyklos.

[7] Devi P.U., Agrawala P.K. (2011). Normal tissue protectors against radiation injury. Def. Sci. J..

[8] Citrin D., Cotrim A.P., Hyodo F., Baum B.J., Krishna M.C., Mitchell J.B. (2010). Radioprotectors and mitigators of radiation-induced normal tissue injury. Oncologist.

[9] Hosseinimehr S.J. (2007). Trends in the development of radioprotective agents. Drug Discov. Today.

[10] Rosen E.M., Day R., Singh V.K. (2015). New approaches to radiation protection. Front. Oncol..

[11] Middleton E. (1998). Effect of plant flavonoids on immune and inflammatory cell function. Flavonoids in the Living System.

[12] Nones J., de Sampaio Spohr T.C.L., Gomes F.C.A. (2012). Effects of the flavonoid hesperidin in cerebral cortical progenitors in vitro: Indirect action through astrocytes. Int. J. Dev. Neurosci..

[13] Yahyapour R., Shabeeb D., Cheki M., Musa A.E., Farhood B., Rezaeyan A., Amini P., Fallah H., Najafi M. (2018). Radiation Protection and Mitigation by Natural Antioxidants and Flavonoids: Implications to Radiotherapy and Radiation Disasters. Curr. Mol. Pharmacol..

[14] Yamada M., Tanabe F., Arai N., Mitsuzumi H., Miwa Y., Kubota M., Chaen H., Kibata M. (2006). Bioavailability of glucosyl hesperidin in rats. Biosci. Biotechnol. Biochem..

[15] Cho J. (2006). Antioxidant and neuroprotective effects of hesperidin and its aglycone hesperetin. Arch. Pharm. Res..

[16] Roohbakhsh A., Parhiz H., Soltani F., Rezaee R., Iranshahi M. (2015). Molecular mechanisms behind the biological effects of hesperidin and hesperetin for the prevention of cancer and cardiovascular diseases. Life Sci..

[17] Garg A., Garg S., Zaneveld L., Singla A. (2001). Chemistry and pharmacology of the citrus bioflavonoid hesperidin. Phytother. Res..

[18] Moher D., Liberati A., Tetzlaff J., Altman D.G. (2009). Preferred reporting items for systematic reviews and meta-analyses: The PRISMA statement. Ann. Intern. Med..

[19] Katoch O., Kaushik S., Kumar M.S.Y., Agrawala P.K., Misra K. (2012). Radioprotective property of an aqueous extract from *Valeriana wallichii*. J. Pharm. Bioallied Sci..

[20] Hosseinimehr S.J., Mahmoudzadeh A., Ahmadi A., Mohamadifar S., Akhlaghpoor S. (2009). Radioprotective effects of hesperidin against genotoxicity induced by γ-irradiation in human lymphocytes. Mutagenesis.

[21] Kalpana K., Devipriya N., Srinivasan M., Menon V.P. (2009). Investigation of the radioprotective efficacy of hesperidin against gamma-radiation induced cellular damage in cultured human peripheral blood lymphocytes. Mutat. Res..

[22] Hosseinimehr S.J., Ahmadi A., Beiki D., Habibi E., Mahmoudzadeh A. (2009). Protective effects of hesperidin against genotoxicity induced by 99mTc-MIBI in human cultured lymphocyte cells. Nucl. Med. Biol..

[23] Kang J.A., Yoon S.H., Rho J.K., Jang B.-s., Choi D.S., Lee D.-E., Byun E.-B., Jeon J., Park S.H. (2016). Radioprotective effect of hesperetin against γ-irradiation-induced DNA damage and immune dysfunction in murine splenocytes. Food Sci. Biotechnol..

[24] Jagetia G.C., Rao K. (2018). Acceleration in the Repair and Regenerative Responses by Different Doses of Hesperidin in the Deep Full Thickness Cutaneous Wound of Mice Whole Body Exposed To 6 Gy of γ -Radiation. Nurs. Health Care Int. J..

[25] Jagetia G., Rao K. (2018). Hesperidin treatment abates radiation-induced delay in healing of deep cutaneous excision wound of mice hemi-body exposed to different doses of γ-radiation. Clin. Dermatol. Dermatitis.

[26] Jagetia G., Rao K. (2018). Hesperidin, a citrus bioflavonoid potentiates repair and regeneration of deep dermal excision wounds of mice whole body exposed to different doses of 60 Co γ-radiation. Clin. Dermatol. Dermatitis.

[27] Jagetia G., Mallikarjuna Rao K. (2015). Hesperidin, a citrus bioflavonoid reduces the oxidative stress in the skin of mouse exposed to partial body γ-radiation. Transcriptomics.

[28] Kalpana K.B., Devipriya N., Srinivasan M., Vishwanathan P., Thayalan K., Menon V.P. (2011). Evaluating the radioprotective effect of hesperidin in the liver of Swiss albino mice. Eur. J. Pharmacol..

[29] Lee Y.-R., Jung J.-H., Kim H.-S. (2011). Hesperidin partially restores impaired immune and nutritional function in irradiated mice. J. Med. Food.

[30] Hosseinimehr S., Nemati A. (2006). Radioprotective effects of hesperidin against gamma irradiation in mouse bone marrow cells. Br. J. Radiol..

[31] Haddadi G., Abbaszadeh A., Mosleh-Shirazi M.A., Okhovat M.A., Salajeghe A., Ghorbani Z. (2018). Evaluation of the effect of hesperidin on vascular endothelial growth factor gene expression in rat skin animal models following cobalt-60 gamma irradiation. J. Cancer. Res. Ther..

[32] Haddadi G.H., Rezaeyan A., Mosleh-Shirazi M.A., Hosseinzadeh M., Fardid R., Najafi M., Salajegheh A. (2017). Hesperidin as Radioprotector against Radiation-induced Lung Damage in Rat: A Histopathological Study. J. Med. Phys..

[33] Shaban N.Z., Zahran A.M.A., El-Rashidy F.H., Kodous A.S.A. (2017). Protective role of hesperidin against γ-radiation-induced oxidative stress and apoptosis in rat testis. J. Biol Res. (Thessalon)..

[34] Karimi N., Monfared A.S., Haddadi G.H., Soleymani A., Mohammadi E., Hajian-Tilaki K., Borzoueisileh S. (2017). Radioprotective effect of hesperidin on reducing oxidative stress in the lens tissue of rats. Int. J. Pharm. Investig..

[35] El-Rahman N., El-Dein E., El-Hady A., Soliman S.M. (2016). Effect of Hesperidin on γ-Radiation-and/or Paraquat Herbicide-Induced Biochemical, Hematological and Histopathological Changes in Rats. Pak. J. Zool..

[36] Rezaeyan A., Fardid R., Haddadi G., Takhshid M., Hosseinzadeh M., Najafi M., Salajegheh A. (2016). Evaluating radioprotective effect of hesperidin on acute radiation damage in the lung tissue of rats. J. Biomed. Phys. Eng..

[37] Fardid R., Ghorbani Z., Haddadi G., Behzad-Behbahani A., Arabsolghar R., Kazemi E., Okhovat M., Hosseinimehr S. (2016). Effects of hesperidin as a radio-protector on apoptosis in rat peripheral blood lymphocytes after gamma radiation. J. Biomed. Phys. Eng..

[38] Rezaeyan A., Haddadi G.H., Hosseinzadeh M., Moradi M., Najafi M. (2016). Radioprotective effects of hesperidin on oxidative damages and histopathological changes induced by X-irradiation in rats heart tissue. J. Med. Phys..

[39] Ahmed H.M., Hussein M.A., Alazonee A.S. (2013). Radioprotective effect of hesperidin against gamma-irradiation-induced oxidative stress and biomechanical properties of bone in rats. Life Sci. J..

[40] Pradeep K., Ko K.C., Choi M.H., Kang J.A., Chung Y.J., Park S.H. (2012). Protective effect of hesperidin, a citrus flavanoglycone, against γ-radiation-induced tissue damage in Sprague-Dawley rats. J. Med. Food.

[41] Said U.Z., Saada H.N., Abd-Alla M.S., Elsayed M.E., Amin A.M. (2012). Hesperidin attenuates brain biochemical changes of irradiated rats. Int. J. Radiat. Biol..

[42] Park S.H., Pradeep K., Ko K.C. (2009). Protective effect of hesperidin against γ-radiation induced oxidative stress in Sprague-Dawley rats. Pharm. Biol..

[43] Rodemann H.P., Blaese M.A. (2007). Responses of normal cells to ionizing radiation. Semin. Radiat. Oncol..

[44] Peña L.A., Fuks Z., Koksnick R. (1997). Stress-induced apoptosis and the sphingomyelin pathway. Biochem Pharmacol..

[45] Pena L.A., Fuks Z., Kolesnick R.N. (2000). Radiation-induced apoptosis of endothelial cells in the murine central nervous system: Protection by fibroblast growth factor and sphingomyelinase deficiency. Cancer Res..

[46] Pearce M.S., Salotti J.A., Little M.P., McHugh K., Lee C., Kim K.P., Howe N.L., Ronckers C.M., Rajaraman P., Craft A.W. (2012). Radiation exposure from CT scans in childhood and subsequent risk of leukaemia and brain tumours: A retrospective cohort study. Lancet.

[47] Prise K.M., Saran A. (2011). Concise review: Stem cell effects in radiation risk. Stem Cells.

[48] Ryan J.L. (2012). Ionizing radiation: The good, the bad, and the ugly. J. Investig. Dermatol..

[49] Porock D., Nikoletti S., Kristjanson L. (1999). Management of radiation skin reactions: Literature review and clinical application. Plast. Surg. Nurs..

[50] Yusuf S.W., Venkatesulu B.P., Mahadevan L.S., Krishnan S. (2017). Radiation-induced cardiovascular disease: A clinical perspective. Front. Cardiovasc. Med..

[51] Mathers C.D., Loncar D. (2006). Projections of global mortality and burden of disease from 2002 to 2030. PLoS Med..

[52] Darby S.C., Ewertz M., McGale P., Bennet A.M., Blom-Goldman U., Brønnum D., Correa C., Cutter D., Gagliardi G., Gigante B. (2013). Risk of ischemic heart disease in women after radiotherapy for breast cancer. N. Engl. J. Med..

[53] Ben-David M.A., Elkayam R., Gelernter I., Pfeffer R.M. (2016). Melatonin for prevention of breast radiation dermatitis: A phase II, prospective, double-blind randomized trial. Isr. Med. Assoc. J..

[54] Lozano A., Marruecos J., Rubió-Casadevall J., Farre N., Lopez-Pousa A., Giralt J., Planas I., Cirauqui B., Lanzuela M., Morera R. (2018). Phase II trial of high-dose melatonin oral gel for the prevention and treatment of oral mucositis in H&N cancer patients undergoing chemoradiation (MUCOMEL). J. Clin. Oncol..

